# A Rare Case of Malignant Glomus Tumor of the Esophagus

**DOI:** 10.1155/2013/287078

**Published:** 2013-12-17

**Authors:** Gurvinder Singh Bali, Douglas J. Hartman, Joel B. Haight, Michael K. Gibson

**Affiliations:** ^1^University Hospitals Case Medical Center, Seidman Cancer Center, Case Western Reserve University, 11100 Euclid Avenue, LKS 5079, Cleveland, OH 44106, USA; ^2^Division of Anatomic Pathology, Department of Pathology, University of Pittsburgh Medical Center, 200 Lothrop Street A610, Pittsburgh, PA 15213, USA; ^3^Penn State Hershey Medical Group Colonnade, 32 Colonnade Way, State College, PA 16803, USA

## Abstract

Glomus tumors are rare neoplasms that usually occur on the hands in a subungual location, or sometimes in palms, wrists or soles of the feet. They are described as purple/pink tiny painful lesions with a triad of pain, local point tenderness, and cold hypersensitivity. They are almost always benign, but rare malignant variants have been reported. They have also been reported to be present at unusual locations, like the lung, stomach, or liver. Gastrointestinal glomus tumors are extremely rare tumors and very few cases have been reported in the literature. Most that have been reported were usually benign in nature. A rare esophageal glomangioma, mimicking a papilloma, was reported in 2006. We report a case of glomangiosarcoma (malignant glomus tumor) in a 49-year-old female, who presented with symptoms of dysphagia including some spasm and hoarseness and subjective unintentional weight loss. On endoscopic exam, she was found to have a distal esophageal mass with malignant features. Radiologically, the mass had a size of about 8 cm on the CT scan without evidence of metastases. Pathology and immunostaining of the biopsy showed features resembling a malignant glomus tumor. She underwent an endoscopic and laparoscopic staging of the tumor along with ultrasound. Based on the laparoscopic findings, which were consistent with the preoperative diagnosis, she was scheduled for an esophagectomy. Histopathology and immunophenotypic features of the excised mass were consistent with a diagnosis of malignant glomus tumor.

## 1. Introduction

Glomus tumors are rare, mostly benign, vascular hamartomatous derivatives of glomus bodies, which are present at arteriovenous anastomoses (without a capillary bed in between), normally located in the dermis. The glomus bodies play a role in thermoregulation of the skin. Glomus tumors usually arise in subungual areas, palms, wrist, and soles of feet as these areas are rich in glomus bodies. They usually present clinically as a triad of severe subungual pain, tenderness localized over a point, and cold hypersensitivity [1, 2]. Glomus tumor at a site other than the limbs is an extremely uncommon finding. They have been reported in noncutaneous locations, such as the lung, stomach, or liver [3–6]. Some exceptionally rare cases of visceral glomus tumors have been reported, most commonly in the stomach. These tumors are almost always benign [7, 8]. A case report of an esophageal glomagioma mimicking a papilloma was reported in 2006 [9]. A case report from 1991 describes a malignant glomus tumor of the mediastinum involving the esophagus while a recent case report describes a case (similar to the case we present here) of malignant glomus tumor of the esophagus with lymph node metastases [10, 11].

## 2. Case Report 

Our patient, a 49-year old female, presented with a history of dysphagia for seven months with subjective unintentional weight loss. Endoscopy with esophageal ultrasound revealed a mass in the distal third of the esophagus with malignant features including ulceration, thick size, and granulation tissue but no mediastinal, celiac, or periportal lymphadenopathy. She also underwent an EUS-guided cold-forceps biopsy of the mass. The initial impression was that of a gastrointestinal stromal tumor (GIST). However, an outside pathology report of the biopsied mass showed that the tumor comprised of smooth muscle cells and was C-Kit (CD117) negative. The final diagnosis of the mass was inconclusive about the nature of the tumor, although a smooth muscle component in the mass was noted. Her original diagnostic material from an outside institution was reviewed. The neoplastic cells were epithelioid with focal spindle cells and necrosis and showed Bcl-2, and CD138 positivity, positivity for Vimentin and Actin, and negative for AE1/3, CAM5.2, CK20, CD56, Desmin, S 100, MPO, CD20, CD21, CD99, CD30, DOG1, and PLAP by immunostains (see Table 1). Immunostaining supported a poorly differentiated neoplasm with smooth muscle differentiation (no evidence of carcinoma). Malignant glomus tumor, synovial sarcoma, leiomyoma, and leiomyosarcoma were the differential diagnoses for this mass.

CT scan of the chest revealed a large heterogenous mass located in the distal third of the esophagus, arising from the posterior wall, measuring 7.6 × 4.9 × 4.2 cm. There were multiple areas of decreased attenuation within the mass. No pulmonary nodules were identified. CT scan of the abdomen and pelvis revealed multiple liver lesions which were hyper-vascular, largest measuring 4 cm, in the left lobe, showing a benign central scar, suggestive of focal nodular hyperplasia, which is a common benign tumor of the liver. No distal para-esophageal or gastrohepatic lymphadenopathy was noticed.

She was then scheduled for a laparoscopic staging with possible biopsy of the mass and US of the liver with biopsy of the nodular lesion the following week. An esophagectomy with regional lymph node dissection was recommended. During the laparoscopic procedure, she underwent an intraoperative biopsy of the esophageal mass and ultrasound (US) of the liver with biopsy of the nodular lesion. During her exploratory laparoscopy, she had no evidence of peritoneal, omental, or hepatic metastasis. EGD showed a nonobstructing esophageal tumor, 35–40 cm from the incisors. Lymph node biopsy of the left gastric node and perigastric node showed no features of malignancy on frozen section. Pathology of the esophageal tumor showed reactive squamous epithelium with no neoplasia at 36 cm of the esophagus. Liver biopsy also turned out to be benign.

**Figure 1:**
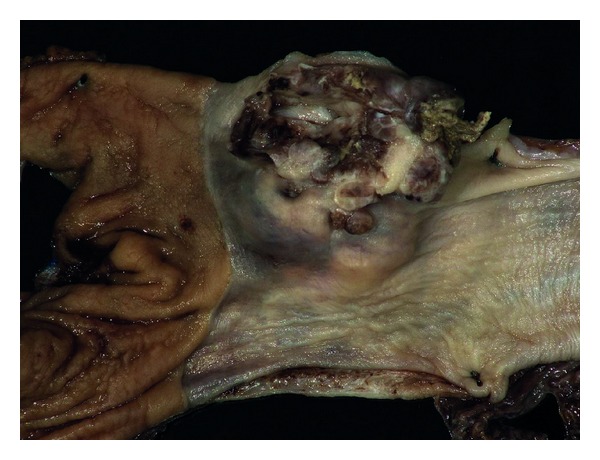
Gross image of the fixed esophagogastrectomy specimen.

**Figure 2:**
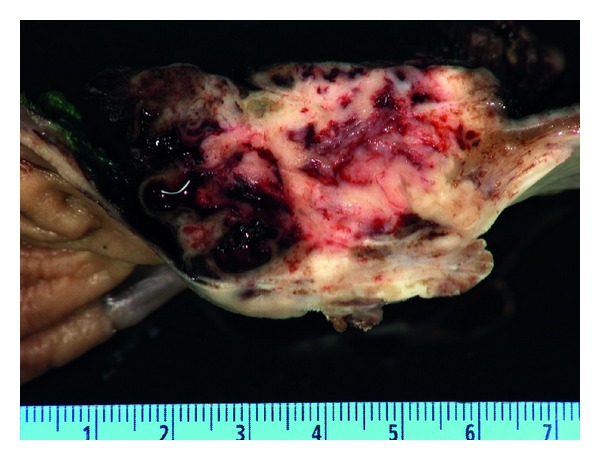
Cross-section of the fixed mass within the esophagus demonstrating the blood spaces and that the lesion is centered on the muscularis propria.

**Figure 3:**
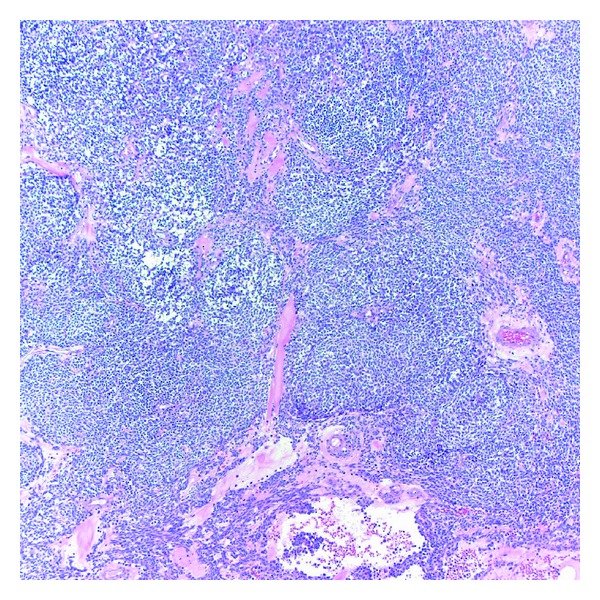
Medium power microscopic image demonstrating a diffuse sheet of medium-sized round blue cells within intermingled vessels (Hematoxylin and Eosin, 100x).

**Figure 4:**
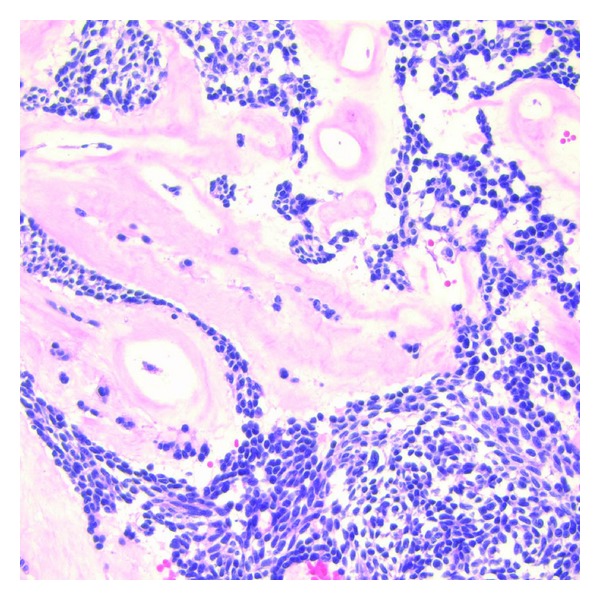
High power microscopic image demonstrating dense hyalinized stroma suggestive of precursor glomus tumor (Hematoxylin and Eosin, 400x).

**Figure 5:**
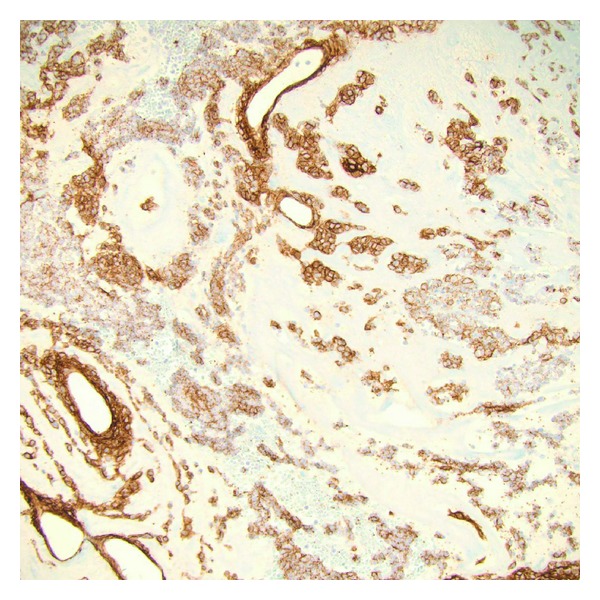
High power microscopic image of a Collagen Type IV immunostain demonstrating pericellular net-like staining around the round blue cells (Collagen Type IV immunostain, 200x).

Surgical excision of the tumor was planned. On a preoperative chest X-ray, the tumor appeared as a 6 × 3 cm oblong mass with smooth margins in the lower left middle-posterior mediastinum. She underwent minimally invasive esophagectomy with partial gastrectomy with lymph node dissection. An esophagogastrectomy specimen containing a 5.0 × 4.5 × 3 cm mass lesion in the distal esophagus, 0.4 cm proximal to the gastroesophageal junction and involving 60% of the circumference of the lumen (Figure 1). The cut surface of this lesion showed lobular, tan, firm mass with prominent blood spaces (Figure 2). The mass was centered on the muscularis propria, ulcerating the mucosal surface and extending into the adventitial soft tissue. The proximal and distal mucosal margins were uninvolved. Microscopically, the neoplastic cells demonstrated a variable morphology of clear cells, plasmacytoid, and spindle cells with occasional blood filled spaces. The predominant histology of the neoplastic cells was a small round blue cell (Figure 3) but in focal areas the neoplastic cells are invested by a dense hyalinized stroma consistent with a glomus tumor (Figure 4). Nine mitotic figures per fifty high power fields were identified. The neoplastic cells are positive for vimentin, actin, calponin, Bcl-2, pericellular net-like collagen IV (Figure 5) by immunostains. The neoplastic cells are negative for DOG1, c-Kit, S100, chromogranin, desmin, CD56, estrogen receptor, CAM5.2, myogenin, WT1, and CD10 by immunostains. The neoplastic cells demonstrate patchy synaptophysin, focal EMA, focal pankeratin and focal NSE. The tumor was negative for the translocation, CIC-DUX4 t(4; 19), arguing against the tumor representing a round cell sarcoma. Additional studies for EWS translocation and SYT translocation were not identified. Given the actin, calponin, pericellular net-like collagen IV positivity, and keratin negativity, this mass likely represents a malignant glomus tumor. These results are summarized in Table 1. There was no evidence of angiolymphatic or perineural invasion. Paraesophageal lymph nodes were also negative for malignancy. A total of 31 nodes were resected, showed no signs of malignancy.

After-surgery, the patient recovered very well and had no complications. Moreover, the tumor appeared to be somewhat of low grade given that there was no perineural and vascular invasion. Surgical excision was carried out with clear margins. Adjuvant radiotherapy or chemotherapy was considered after the surgery, but the risks and complications of radiotherapy like stricture and aspiration, nausea, vomiting, and cramping outweighed the benefits and there was no evidence to prove that adjuvant chemotherapy was linked to improvement in survival. The patient was to be followed up with scans every three months. On her follow-up visit at six months after surgery, her appetite had improved, dysphagia subsided, and she has also gained weight. The patient is doing remarkably well. There is no evidence of recurrence or metastatic disease on her latest CT scan.

This variety of tumor was a surprising diagnosis in this female, who was being followed up for history of dysphagia and subjective weight loss, found to have a distal esophageal mass eroding into the lumen with possible granulation tissue on an EGD.

## 3. Discussion 

Glomus bodies are mostly found in the dermis or subcutis of fingers or palms, or toes and soles of the feet. These play a role in regulating blood flow and temperature. Benign glomus tumors resemble the normal glomus body, as vascular, hamartomatous derivatives of it. Glomus tumors are usually found in the extremities, which is also the most common location of Glomus body. They account for about 1% of hand tumors in adults, usually in females. Glomus tumors are almost always tumors of adults and apparently have no association with other diseases. They may, as a rare exception, present as glomangiosarcomas, which is a malignant variant. It is supposed to arise de novo, as a low-grade malignancy, and presents with same features but is characterized by local recurrence after resection with or without metastasis.

Extradigital sites are seldom seen with glomus tumors, and a malignant glomus tumor of the esophagus, moreover, is an exceptional occurrence. There have been some small case series describing glomus tumors of the antrum of the stomach. Malignant glomus tumor of the esophagus is a rare entity and our current case represents a unique case due to the lack of lymph node metastases. This 49-year-old female, who presented with 6–8 months of dysphagia, upon endoscopic findings, was diagnosed with a tumor invading and eroding into the lumen with possible granulation tissue around it. The differential diagnosis was that of a gastrointestinal stromal tumor (GIST, being the most common submucosal tumor), a smooth muscle tumor (leiomyoma), sarcoma, neuroendocrine tumor, but the possibility of each one of these was ruled out by histologic features, immunohistochemical staining, and FISH studies. Based on the histologic features of plasmacytoid and spindle cells with occasional blood filled spaces and immunophenotype by actin and collagen Type 4, a diagnosis of malignant glomus tumor was made.

The management of this localized mass without metastasis was surgical resection with follow-up with scans. No adjuvant chemotherapy/radiotherapy was given in this case. From a clinical and radiological standpoint, a differential diagnosis of a rare glomus tumor must be considered if the neoplasm is solitary, vascular, and localized (which is usually the case with glomus tumors), without lymph node or distant metastasis. The cornerstone of diagnosis is pathological features and immunostaining. The prognosis is not bad if the disease is localized. However, a close watch with regular scans should be kept for the possibility of recurrence, which is a feature of malignant glomus tumors. Postsurgical resection, adjuvant radiotherapy, or chemotherapy has a limited proven documented role, as the tumor is one of the rare kinds of gastrointestinal malignancies. Risks and complications of radiotherapy are more concerning and disabling than any proven benefits.

## Figures and Tables

**Table 1:** Immunohistochemical/FISH results on specimens.

	Mucosal Biopsy	Surgical Resection
Actin	Positive	Positive
Vimentin	Positive	Positive
Bcl-2	Positive	Positive
CD138	Positive	Not done
Calponin	Not done	Positive
Collagen type IV	Not done	Pericellular net-like pattern
EMA	Not done	Focal Positive
AE1/3	Negative	Focal Positive
Cam5.2	Negative	Negative
CK20	Negative	Not done
CD56	Negative	Negative
Desmin	Negative	Negative
S100	Negative	Negative
MPO	Negative	Not done
CD20	Negative	Not done
CD21	Negative	Not done
CD99	Negative	Not done
CD30	Negative	Not done
C-Kit	Negative	Negative
DOG1	Negative	Negative
PLAP	Negative	Not done
WT1	Not done	Negative
CD10	Not done	Negative
Chromogranin	Not done	Negative
Synaptophysin	Not done	Patchy Positive
NSE	Not done	Focal Positive
Myogenin	Not done	Negative
EWS translocation	Not done	Negative
SYT translocation	Not done	Negative
CIC-DUX4 translocation	Not done	Negative
